# Technical efficiency and productivity of Chinese county hospitals: an exploratory study in Henan province, China

**DOI:** 10.1136/bmjopen-2014-007267

**Published:** 2015-09-09

**Authors:** Zhaohui Cheng, Hongbing Tao, Miao Cai, Haifeng Lin, Xiaojun Lin, Qin Shu, Ru-ning Zhang

**Affiliations:** Department of Health Management, School of Medicine and Health Management, Tongji Medical College, Huazhong University of Science and Technology, Wuhan, Hubei province, People's Republic of China

## Abstract

**Objectives:**

Chinese county hospitals have been excessively enlarging their scale during the healthcare reform since 2009. The purpose of this paper is to examine the technical efficiency and productivity of county hospitals during the reform process, and to determine whether, and how, efficiency is affected by various factors.

**Setting and participants:**

114 sample county hospitals were selected from Henan province, China, from 2010 to 2012.

**Outcome measures:**

Data envelopment analysis was employed to estimate the technical and scale efficiency of sample hospitals. The Malmquist index was used to calculate productivity changes over time. Tobit regression was used to regress against 4 environmental factors and 5 institutional factors that affected the technical efficiency.

**Results:**

(1) 112 (98.2%), 112 (98.2%) and 104 (91.2%) of the 114 sample hospitals ran inefficiently in 2010, 2011 and 2012, with average technical efficiency of 0.697, 0.748 and 0.790, respectively. (2) On average, during 2010–2012, productivity of sample county hospitals increased by 7.8%, which was produced by the progress in technical efficiency changes and technological changes of 0.9% and 6.8%, respectively. (3) Tobit regression analysis indicated that government subsidy, hospital size with above 618 beds and average length of stay assumed a negative sign with technical efficiency; bed occupancy rate, ratio of beds to nurses and ratio of nurses to physicians assumed a positive sign with technical efficiency.

**Conclusions:**

There was considerable space for technical efﬁciency improvement in Henan county hospitals. During 2010–2012, sample hospitals experienced productivity progress; however, the adverse change in pure technical efficiency should be emphasised. Moreover, according to the Tobit results, policy interventions that strictly supervise hospital bed scale, shorten the average length of stay and coordinate the proportion among physicians, nurses and beds, would benefit hospital efficiency.

**Strengths and limitations of this study**

- This study not only evaluated the technical efficiency and productivity of county hospitals in Henan province, China, during the period of healthcare reform, but also explored factors impacting on technical efficiency, which have not been analysed before.
- The study provided an insight into the performance of county hospitals during the reform process in China, which can assist policymakers in choosing the best regulatory framework for the ongoing hospital reform process.
- This study was not able to collect information about case-mix index or patient outcome quality of each hospital due to the dated hospital information system.
- County hospitals in other regions of China were not included in this study.

## Introduction

As China is the most populous country in the world, its healthcare system, potentially affecting the lives of 1.36 billion people, is of concern to researchers all over the world. The healthcare system in China consists of community health centres, and secondary and tertiary hospitals, in the urban areas, while rural areas have village clinics, township health centres and county hospitals. Hospitals are classiﬁed into three levels: tertiary, secondary and primary. Tertiary hospitals have more than 500 beds, treat complicated diseases and provide specialised care. Secondary hospitals have 100–499 beds and treat common illnesses, while primary hospitals have 20–99 beds, and provide preventive and basic medical services.^1^ Among the health service providers in the healthcare system, county hospitals play a crucial role. They serve as the leader of the three-tier healthcare network in rural areas, and connect patients in the village to urban tertiary hospitals.^2^ ^3^ In 2012, the number of county hospitals reached 10 940, accounting for 47.22% of the total number of hospitals, covering more than 900 million people, and providing medical services to approximately 70% of county residents.^4^ Their performance has a signiﬁcant effect on the well-being of the Chinese people. In this paper, we examine county hospitals.

China has achieved an economic miracle since the economic reforms in 1978. However, the development of efficiency and equity in healthcare has for long lagged behind.^5^ Healthcare expenditure has been growing rapidly, but the proportion of the population with access to healthcare has diminished, widening health gaps between urban and rural areas.^6–8^ In response to these inequities, the government of China launched the Healthcare Reform Plan in 2009, and promised to build a safe, effective, convenient and inexpensive universal healthcare network. The plan has ﬁve key tasks:^9^ (1) expanding the coverage of health insurance; (2) establishing an essential drug distribution system; (3) strengthening primary care facilities; (4) promoting equitable access to public health services; and (5) piloting reform of public hospitals. Substantial positive results have been achieved from the reform. For instance, health insurance coverage has been expanded to cover more than 96% of residents; primary healthcare networks have been strengthened, in particular, 2200 county hospitals have been rebuilt or upgraded.^10^ Along with the expansion of health insurance coverage and the strengthening of county hospitals, people's health demands were quickly increasing. A number of county hospitals have increased their beds scale to meet the rising demands. During 2009–2013, the number of beds in county hospitals expanded from 765 510 to 1 238 500, and the total number of inpatients increased from 26 228 716 to 44 565 675.^11^ Technical efficiency (TE) of county hospitals, trend of productivity and factors affecting efficiency are all important considerations under these conditions.

There has been an extensive body of literature dealing with the efficiency of healthcare, and data envelopment analysis (DEA) is the best known methodology and is widely used. Hollingsworth *et al*^12^ systematically reviewed DEA articles on healthcare, involving a wide range of applications. Barnum *et al*^13^ employed DEA to measure the efficiency of hospital pharmacies in America. Dimas *et al*^14^ evaluated the productivity of 22 Greek public hospitals, and found that productivity changes were dominated by technical change. Zere *et al*^15^ measured the TE and productivity of 86 hospitals in South Africa, and examined the impact of hospital characteristics on efficiency and productivity, using Tobit and Ordinary Least Square (OLS) regression. Tlotlego *et al*^16^ adopted the Malmquist index to analyse the productivity of 21 non-teaching hospitals in Botswana during 2006–2008, and found significant inefficiencies. In China, Ng^17^ evaluated the productivity of hospitals in Guangdong province, and found that productivity growth was deteriorating as technology progress. Li *et al*^18^ researched the productivity of 12 tertiary hospitals in Beijing during 2006–2009, and found that sample hospitals were experiencing productivity growth with technological changes (TCs).

In this paper, we focused on the performance of county hospitals in Henan province, China. Henan, with 105.43 million people, is the most populous province in China, and 57.6% of this population live in rural areas. The total health expenditure in Henan province was US$23.3 billion, accounting for 5.13% of its gross domestic product (GDP).^19^ County hospitals play a crucial role in Henan's healthcare system. During 2010–2012, county hospitals developed significantly: the number of beds in county hospitals increased by 11.34%, reaching 78 696, and accounting for 62.81% of the total number of secondary hospitals.^20^ In addition, more than 15.64% of the total expenditure on health was spent in county hospitals.^19^ Though much has changed in Henan county hospitals, little is known about their performance. Thus, the overall objectives of this paper were: (1) to measure the TE of Henan county hospitals during the reform process from 2010 to 2012; (2) to evaluate changes in productivity during the reform; (3) to determine whether, and how, TE is affected by various factors.

## Methods

### Efficiency evaluation methods

To measure the efﬁciency of healthcare organisations, two frontier methodologies, stochastic frontier analysis (SFA) and DEA, have been widely applied.^21–24^ In comparison with DEA, SFA requires constructing an efficient frontier function, and the information of input prices, output prices and total costs is difficult to acquire.^25^ ^26^ Thus, considering the convenience and the multiple inputs and outputs, we applied DEA in this paper.

### Data envelopment analysis

Charnes, Cooper and Rhodes (CCR), Banker, Charnes, Cooper (BCC) and Malmquist productivity index, as models of DEA, have been widely used to measure relative efficiency and productivity. The CCR model, which was proposed by Charnes *et al*,^27^ assumes that production is constant return to scale (CRS), which means an increase in the input(s) will result in a proportionate increase in the output(s). When a hospital is operating at CRS, TE is equal to scale efficiency (SE). However, when Decision Making Units (DMUs) are not operating at optimum scale, TE measured with the CCR model may be altered by SE. The BCC model, which was proposed by Banker *et al*,^28^ assumes that production is variable return to scale (VRS), which means an increase in the input(s) will result in either an increase or a decrease in the output(s). VRS has two dimensions: increasing returns to scale (IRS), which means 1% increase in inputs will be followed by more than 1% increase in outputs, and decreasing returns to scale (DRS), which means 1% increase in inputs will result in less than 1% increase in outputs. The BCC model can incorporate the impact of SE in measurement of TE.^29^
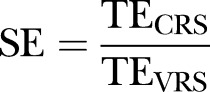


The Malmquist productivity index, developed by Färe *et al*,^30^ can take into account the productivity changes of hospitals in a time-series setting. Total factor productivity change (TFPC) can be decomposed into TE changes (TECs) and TCs. In the Malmquist productivity index, five indices can be generated: TFPC, TEC, TC, pure TEC (PTEC) and SE change (SEC).^18^ ^31^
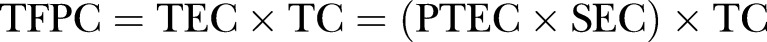


### Input orientation

In this paper, an input-oriented DEA was employed for the following reasons. First, due to the ineffectiveness of the tiered Chinese diagnosis and treatment system, the selection of hospitals depends on patients, which makes it difficult for hospitals to estimate the actual healthcare demand. Second, the input orientation reflects the regulated context of Chinese county hospitals, that hospital managers have more control over their inputs (staffing and operating expenses or beds) than they do over outputs (patients get admitted or visit the outpatient department). Third, the input orientation is more consistent with the functional orientation of county hospitals in China, which is obliged to meet people's basic and commonest health demands with reducing or limiting input use. In addition, the selection of input orientation is also conformed to the character of the non-proﬁt organisation, which focuses on the minimisation of inputs with given outputs, and the selection is consistent with other empirical research regarding hospital efficiency evaluation.^17^ ^23^ ^32^

### Tobit regression analysis

In this paper, the Tobit regression model was employed to relate the TE_CRS_ to a number of explanatory variables. Considering that the efficiency scores fall between 0 and 1, and several scores tend to concentrate on these boundary values (ie, censored at 1), ordinary least squares are inapplicable.^33^ Therefore, the Tobit model was applied.^34^ For convenient computation, in the Tobit model, a censoring point is preferable to assume at zero, so that fully efficient hospitals could be constrained at 0 whereas the inefficient hospitals showed scores greater than 0. Following Asbu,^35^ the TE_CRS_ scores were transformed into inefficiency scores and left censored at 0 using the formula:
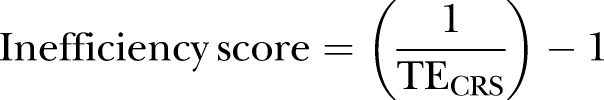


This transformation of the dependent variable also reversed the signs of the coefficient in the regression.

## Data

The sample county hospitals in this study were selected from Henan province, China. Data were obtained from the National Health Statistical Information Report System during 2010–2012. Since the basic requirement in applying DEA is to select a group of similar units, the samples were carefully scrutinised to eliminate errors. First, to reduce differences in disease complexity, and in medical technology and quality of care among hospitals, only public county general hospitals were selected. Second, hospitals with the data of default values or abnormal values were excluded. Third, hospitals without data for three consecutive years were excluded to ensure consistency. Finally, 114 public county general hospitals in Henan province were selected in the data set.

## Variables selection

### Input and output variables

In this paper, the selection of input and output variables were guided by previous empirical studies,^17^ ^18^ ^36–38^ and depended on the availability of data in the National Health Statistical Information Report System.

Regarding the input variables, both labour and capital were considered important in delivering health services. In this paper, the labour variables focused on two indicators: ‘the number of physicians’ and ‘the number of nurses’. Regarding capital, most studies considered ‘the number of beds’ as a proxy of capital inputs. Owing to the dynamic situation of hospitalisation in China, characterised by the use of many temporary beds, ‘actual number of open beds’ instead of ‘the number of beds’, was considered.

Regarding the output variables, following the hospital efficiency studies conducted by Tlotlego *et al*^16^ and Li *et al*,^18^ hospital outputs in this study were represented by ‘the number of outpatient and emergency visits’ and ‘the number of inpatient days’. Since inpatient health services have different features and consume more resources than outpatient services, the use of ‘inpatient days’ was more medically homogeneous than the ‘inpatient’ variable and can provide a more favourable hospital output.

The output variables in this study are all aggregated; however, hospitals provide services to patients who differ in terms of case-mix variation and quality. Since there was no available data to accurately measure the case-mix indices based on diagnosis-related groups, the use of case-mixed index was limited for hospitals in China as well as in other developing countries.^39^

The descriptive analysis of the inputs and outputs was conducted using SPSS V.13.0 statistical software. TE and productivity were computed using DEAP V.2.1 software.^40^

### Explanatory variables

In the Tobit regression analysis, the technical inefficiency scores were regressed against a set of environmental characteristics and several institutional factors.

Following literature review, factors that influence hospital efficiency were classified. Environmental factors include: GDP/GDP per capita, catchment population, geographic location, market structure/competition and government subsidy. Institutional factors include: ownership, hospital size/capacity, output quality, teaching status, bed occupancy rate (OCCU), average length of stay (ALoS), ratio of nurses to physicians (RONTP) and ratio of beds to nurses (ROBTN).^21^ ^38^ ^41–43^ Considering the samples in this study were public county general hospitals from Henan and relevant data regarding quality was unavailable, the environmental factor of location and institutional factors of ownership, output quality and teaching status, were excluded.

Thus, four environmental variables including ‘catchment population’, ‘GDP per capita’, ‘Herfindahl-Hirschman Index (HHI)’ and ‘government subsidy’, as well as five institutional variables including ‘hospital beds group’, ‘ALoS’, ‘OCCU’, ‘RONTP’ and ‘ROBTN’, were selected for inclusion in this study. The estimated Tobit model was as follows:
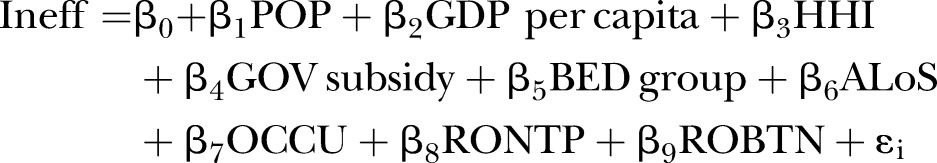


Where Ineff is the technical inefficiency score, POP is the region catchment population dummy variable (0=if population is less than 500 000; 1=if population is 500 000 and above); HHI is a variable that describes market competition; BED group is the hospital beds dummy variable, beds were stratified according to quartiles (size 1=hospitals with 100–227 beds; size 2=hospitals with 228–445 beds; size 3=hospitals with 446–617 beds; size 4=hospitals with more than 618 beds); and ε_i_ is the error term. Tobit regression was conducted using STATA V.11.0 statistical software.^44^

## Results

The descriptive statistics of inputs, outputs and explanatory variables are shown in table 1.

**Table 1:** Descriptive statistics of inputs, outputs and explanatory variables

	2010	2011	2012		2010	2011	2012
*Inputs*				*Outputs*			
Physicians				Outpatient and emergency visits			
Mean	112	114	122	Mean	170 008	190 256	218 887
Maximum	256	237	344	Maximum	498 574	583 348	593 502
Minimum	23	20	20	Minimum	26 280	28 291	34 920
SD	51	55	62	SD	94 413	103 403	115 943
Nurses				Inpatient Days			
Mean	153	174	194	Mean	112 391	127 401	152 491
Maximum	430	453	575	Maximum	298 062	342 384	390 013
Minimum	27	26	23	Minimum	15 862	23 250	23 253
SD	80	95	110	SD	64 341	74 159	88 382
Hospital beds							
Mean	373	396	462				
Maximum	915	1109	1109				
Minimum	100	100	100				
SD	193	213	243				
*Explanatory variables*							
GDP per capita				ALoS			
Mean	24 957	29 084	31 236	Mean	7.8	7.9	7.9
Maximum	70 006	70 473	74 571	Maximum	14.5	14.6	14.6
Minimum	9282	11 531	13 975	Minimum	4.7	5.0	4.1
SD	13 249	14 86	15 808	SD	1.6	1.6	1.6
Catchment population (POP)				OCCU			
Mean	660 000	650 000	650 000	Mean	85.0%	90.4%	92.5%
Maximum	160 000	1 630 000	1 630 000	Maximum	115.6%	131.2%	149.0%
Minimum	70 000	70 000	70 000	Minimum	33.2%	42.2%	43.1%
SD	270 000	270 000	260 000	SD	16.2%	14.9%	14.8%
HHI				RONTP			
Mean	504	519	524	Mean	1.37	1.56	1.63
Max	1278	1287	1310	Max	2.27	3.90	4.30
Min	288	286	285	Min	0.57	0.50	0.46
SD	168	170	172	SD	0.36	0.52	0.60
Proportion of government subsidy in hospital revenues (GOV subsidy)				ROBTN			
Mean	4.59%	5.99%	5.84%	Mean	2.60	2.48	2.69
Maximum	50.11%	43.51%	78.92%	Maximum	5.56	6.84	8.87
Minimum	0.00%	0.00%	0.00%	Minimum	1.21	1.29	1.02
SD	7.65%	7.78%	10.18%	SD	0.84	0.93	1.29

ALoS, average length of stay; GDP, gross domestic product; HHI, Herfindahl-Hirschman Index; OCCU, bed occupancy rate; POP, regional catchment population dummy variable; ROBTN, ratio of beds to nurses; RONTP, ratio of nurses to physicians.

The TE and SE of sample county hospitals during 2010–2012 are shown in table 2.

**Table 2:** Technical and scale efficiency of hospitals, and frequency distribution during 2010–2012

	2010			2011			2012		
	TE_CRS_	TE_VRS_	SE	TE_CRS_	TE_VRS_	SE	TE_CRS_	TE_VRS_	SE
Mean	0.697	0.751	0.932	0.748	0.789	0.949	0.790	0.816	0.969
Median	0.722	0.741	0.984	0.749	0.777	0.990	0.788	0.812	0.992
Maximum	1.000	1.000	1.000	1.000	1.000	1.000	1.000	1.000	1.000
Minimum	0.297	0.492	0.441	0.353	0.564	0.536	0.482	0.518	0.721
SD	0.129	0.112	0.122	0.123	0.112	0.091	0.121	0.119	0.056
Hospital ranking									
100%	2 (1.8%)	6 (5.3%)	6 (5.3%)	2 (1.8%)	9 (7.9%)	9 (7.9%)	10 (8.8%)	18 (15.8%)	17 (14.9%)
80–99.9%	22 (19.3%)	31 (27.2%)	94 (82.4%)	39 (34.2%)	40 (35.1%)	97 (85.1%)	46 (40.3%)	46 (40.4%)	92 (80.7%)
60–79.9%	63 (55.3%)	68 (59.6%)	14 (12.3%)	61 (53.5%)	62 (54.4%)	8 (7.0%)	53 (46.5%)	47 (41.2%)	5 (4.4%)
40–59.9%	24 (21.0%)	9 (7.9%)	0 (0.0%)	10 (8.8%)	3 (2.6%)	0 (0.0%)	5 (4.4%)	3 (2.6%)	0 (0.0%)
<40%	3 (2.6%)	0 (0.0%)	0 (0.0%)	2 (1.7%)	0 (0.0%)	0 (0.0%)	0 (0.0%)	0 (0.0%)	0 (0.0%)

CRS, constant return to scale; DEA, data envelopment analysis; SE, scale efficiency=TE_CRS_/TE_VRS_; TE_CRS_, overall technical efficiency from CRS DEA; TE_VRS_, pure technical efficiency from VRS DEA; VRS, variable return to scale.

### Overall TE (TE_CRS_)

For the years 2010, 2011 and 2012, out of the 114 hospitals, 2 (1.8%), 2 (1.8%) and 10 (8.8%) hospitals, respectively, were defined as technically efficient. While, 112 (98.2%), 112 (98.2%) and 104 (91.2%) hospitals were inefficient, respectively. Average TE_CRS_ was 0.697, 0.748 and 0.790, respectively.

### Pure TE (TE_VRS_)

In 2010, 2011 and 2012, 6 (5.3%), 9 (7.9%) and 18 (15.8%) hospitals, respectively, operated at the best efficiency levels, with a TE_VRS_ score of 1.000. In total, 108 (94.7%), 105 (92.1%) and 96 (84.2%) hospitals, respectively, operated inefficiently. Average TE_VRS_ was 0.751, 0.789 and 0.816, respectively, implying that if they run efficiently, the hospitals should decrease 24.9%, 21.1% and 18.4% of inputs for the same volume of outputs.

### Scale efficiency

As shown in table 2, for the years 2010, 2011 and 2012, average SE was 0.932, 0.949 and 0.969, respectively.

Six (5.3%), 11 (9.7%) and 19 (16.7%) hospitals manifested CRS, indicating that they operated at their most productive size. One hundred (87.7%), 96 (84.2%) and 83 (72.8%) showed IRS, suggesting that they should expand their scale to become scale efficient. Eight (7.0%), 7 (6.1%) and 12 (10.5%) hospitals experienced DRS, meaning that they should scale down to become scale efficient. Average number of beds in IRS, CRS and DRS hospitals, respectively, was 366, 537 and 701. Hospitals of DRS had more beds than those of IRS and CRS (Mann-Whitney U test Z=−3.065, p=0.002; Mann-Whitney U test Z=−6.692, p=0.000).

### Potential input reductions

In order to make inefficient county hospitals operate efficiently, there would be potential for significant input savings. Table 3 presented the total inputs that needed reductions. In 2012, for example, the inefficient hospitals combined would need to reduce the number of physicians, nurses and beds by 3417 (24.54%), 5029 (22.70%) and 10 233 (19.44%), respectively.

**Table 3:** Total input reductions needed to make hospitals efficient

	2010			2011			2012		
Inputs	Actual values	Target values	Difference (%)	Actual values	Target values	Difference (%)	Actual values	Target values	Difference (%)
Physicians	12 806	8769	−4037 (−31.53%)	13 006	9527	−3479 (−26.75%)	13 925	10 508	−3417 (−24.54%)
Nurses	17 494	12 717	−4777 (−27.31%)	19 849	15 003	−4846 (−24.42%)	22 153	17 124	−5029 (−22.70%)
Beds	42 476	31 536	−10 940 (−25.75%)	45 146	35 100	−10 046 (−22.25%)	52 627	42 394	−10 233 (−19.44%)

### Productivity growth (TFPC)

The Malmquist productivity index was applied to analyse the changes in productivity over the 2010–2012 period, and the year 2010 has been taken as the technology reference. Table 4 presents the Malmquist index summary of annual geometric means. On average, TFPC increased by 7.8%, among which, TC increased by 6.8% and TEC increased slightly by 0.9%. Thus, the increase of TC was the main contributor for the TFPC improvement. During 2010–2012, 88 (77.2%) hospitals had TFPC scores greater than 1, indicating growth in productivity; 26 (22.8%) hospitals had TFPC scores less than 1, indicating deterioration in productivity.

**Table 4:** Malmquist index summary of annual means (input oriented)

Year	Technical efficiency change (A=(C×D))	Technological change (B)	Pure technical efficiency change (C)	Scale efficiency change (D=(A/C))	Total factor productivity change (E=A×B)
2011	1.029	1.058	0.981	1.049	1.088
2012	0.989	1.079	1.000	0.990	1.067
Mean	1.009	1.068	0.990	1.019	1.078
Frequency distribution (2010–2012)					
>1	56 (49.1%)	114 (100.0%)	43 (37.7%)	66 (57.9%)	88 (77.2%)
1	2 (1.8%)	0 (0.0%)	13 (11.4%)	6 (5.3%)	0 (0.0%)
<1	56 (49.1%)	0 (0.0%)	58 (50.9%)	42 (36.8%)	26 (22.8%)
Frequency distribution (2010–2011)					
>1	59 (51.8%)	112 (98.2%)	39 (34.2%)	90 (78.9%)	85 (74.6%)
1	5 (4.4%)	0 (0.0%)	17 (14.9%)	7 (6.1%)	0 (0.0%)
<1	50 (43.8%)	2 (1.8%)	58 (50.9%)	17 (15.0%)	29 (25.4%)
Frequency distribution (2011–2012)					
>1	50 (43.8%)	114 (100.0%)	50 (43.8%)	30 (26.3%)	81 (71.1%)
1	5 (4.4%)	0 (0.0%)	16 (14.0%)	7 (6.1%)	0 (0.0%)
<1	59 (51.8%)	0 (0.0%)	48 (42.2%)	77 (67.6%)	33 (28.9%)

A score >1 indicates growth; a score of 1 signifies stagnation; a score <1 indicates decline or deterioration.

### Technological change

During 2010–2012, all sample hospitals experienced technical progress. The average TC score was 1.068, indicating a 6.8% technical improvement over the period.

### TE changes

TEC is a product of PTEC and SEC. During 2010–2012, the average TEC was 1.009, for an improvement in SEC of 1.9%, and counterbalanced by the decline in PTEC of 1%. In this study, the average PTEC was 0.990 less than the average SEC of 1.019, meaning that there was an inefficient use of inputs. During 2010–2011 and 2011–2012, average SEC decreased from 1.049 to 0.990, and average PTEC increased from 0.981 to 1.000.

### Tobit regression analysis of impacting factors on technical inefficiency

In this paper, Tobit regression was employed to relate the technical inefficiency scores to four environmental and five institutional variables in 2012, the last year. The results are presented in table 5.

**Table 5:** Result from Tobit regression analysis (N=114, year=2012)

Variable	Coefficient	SE	t-Ratio	p>|t|
POP	−0.028	0.032	−0.88	0.382
GDP per capita	−0.000	0.000	−1.56	0.121
HHI	0.000	0.000	0.70	0.482
GOV subsidy	0.246	0.131	1.89	0.062^*^
Bed group				
Size 2 (228–445)	0.052	0.040	1.29	0.201
Size 3 (446–617)	0.025	0.037	0.67	0.507
Size 4 (>618)	0.112	0.037	3.00	0.003***
ALoS	0.021	0.009	2.34	0.021**
OCCU	−1.294	0.100	−12.92	0.000***
RONTP	−0.109	0.028	−3.76	0.000***
ROBTN	−0.103	0.013	−8.05	0.000***
Constant	1.713	0.137	12.52	0.000***
Sigma	0.131	0.009		
Observations summary	10 left-censored observations at *Ineff≤0*			
	104 uncensored observations			
	0 right-censored observations at *Ineff≥1*			
Number of observations	114 sample county hospitals in 2012			
Log likelihood	55.55			
χ^2^	126.34			
Probability>χ^2^	0.00***			

A negative coefficient indicated a positive association with TE_CRS_ and a positive coefficient meant a negative association with TE_CRS_.

^*^Significant at the 0.10 level, two-tailed test.

^**^Significant at the 0.05 level, two-tailed test.

^***^Significant at the 0.01 level, two-tailed test.

ALoS, average length of stay; GDP, gross domestic product; HHI, Herfindahl-Hirschman Index; OCCU, bed occupancy rate; POP, regional catchment population dummy variable; ROBTN, ratio of beds to nurses; RONTP, ratio of nurses to physicians; TE_CRS_, overall technical efficiency from CRS data envelopment analysis.

Regarding environmental factors, ‘POP’ (p=0.382), ‘GDP per capita’ (p=0.121) and ‘HHI’ (p=0.482) were, respectively, statistically insignificant. However, ‘GOV subsidy’ exhibited a positive and significant sign (p=0.062), indicating that sample hospitals with a higher proportion of government subsidy were possibly technically inefficient.

Regarding institutional factors, ‘BED group of SIZE 4’ was positively associated with technical inefficiency (p=0.000), indicating that sample hospitals with more than 618 beds have lower TE. The ‘ALoS’ was statistically significant (p=0.021) and assumed a positive sign with technical inefficiency. Internal management variables, such as ‘OCCU’, ‘RONTP’ and ‘ROBTN’, assumed negative signs with technical inefficiency and were statistically significant (p<0.001).

## Discussion

The results of this study indicated that sample county hospitals experienced significant technical inefficient during 2010–2012. Only 1.8%, 1.8% and 8.8% of sample hospitals were defined as overall technically efficient, indicating a great need for efficiency improvement, while the average SE in sample hospitals was quite high and increased from 0.932 in 2010 to 0.969 in 2012. Thus, the lower level of pure TE, which progressed slightly from 0.751 to 0.816 over the period, may account for the overall technical inefficiency, while the efficiency of Henan county hospitals was lower than those reported in other provinces of China. For example, the average score of TE, pure TE and SE in county hospitals in Jiangxi was 0.936, 0.978 and 0.960, respectively;^45^ and in Chongqing, during 2008–2011, it was 0.936, 0.978 and 0.960, respectively.^46^ These differences might be attributed to the differences among input-output indicators and external environments. In this paper, we also computed the potential for inputs savings. For example, the excess physicians, nurses and beds from inefficient hospitals, respectively, were 3417, 5029 and 10 233 in 2012. Considering the substantial support from the Chinese government for encouraging the development of private hospitals,^47^ ^48^ and the understaffed primary health facilities,^8^ policymakers might consider reassigning the excess hospital staff and beds to the private hospitals and primary health facilities where they are actually needed.

The results indicated that sample county hospitals were experiencing a TFPC progress during 2010–2012 and substantial TCs were the largest contributors, which was consistent with the findings in Shandong^49^ and Guangdong provinces.^17^ The average TFPC score of 1.078 in Henan county hospitals was also comparable to the scores in other countries, for instance, a score of 0.92 in Montreal, Canada,^50^ 1.028 in regional hospitals in Ireland,^51^ 1.045 in Angolan municipal hospitals^52^ and of 1.209 in Brazilian federal university hospitals.^53^ There were many factors contributing to the TCs, while the adoption of high tech treatments and equipment contributed most in sample hospitals. Ng^17^ indicated the observed productivity growth from 68% to 94% resulted from the adoption of high tech treatments. In China, although the majority of hospitals are publicly owned, government subsidies account for less than 10% of their total revenues; the remaining 90% have to be obtained from revenue-generating activities. In addition, since health services in China have long been underpriced, hospitals have to obtain higher margins from services provided by high tech treatments or high tech equipment, which is a driving factor to increase their number of high tech treatments and equipment. As shown in our results, the TCs had increased each year. Pang and Wang^54^ pointed out that the introduction of new medical devices and the use of new drugs, etc, can promote technological progress in the short term in Chinese hospitals. However, the lack of qualified staff and the inadequate use of medical equipment may reduce the technological efficiency in the long run, a fact that should be given more attention.

TEC insignificantly contributed to growth of the TFPC in the results, which was consistent with the findings of Li *et al*.^18^ The small progress or decline in TEC mainly results from the stagnation and decline of PTEC, which might be attributed to poor hospital governance and management. Despite having an autonomic authority in the generation and management of finances, county hospitals in China are governed by multiple bodies. Strategic hospital decisions are controlled by administrative rules and at least eight government ministries involved in appointment and management of personnel, internal organisation and investment decisions, etc.^55^ Moreover, hospital directors in China are mostly appointed by the local government, without well-designed mechanisms, rules and regulations; thus, the lack of clearly defined rights and responsibility increases their subjectivity in decision-making, which would decrease the quality of management practices and further influence pure TE.^18^ Fang *et al*^4^ summarised the problems among Chinese county hospitals, and put forward the ‘corporate governance model’ to improve hospital governance and management, which may help to improve pure TE in county hospitals. We also noticed that the SEC deteriorated during 2011–2012 after an increase in 2010–2011, which suggests that sample county hospitals should explore their optimum operation scale.

In this study, the Tobit regression results indicated that sample hospitals with a higher proportion of government subsidy in their revenues were assumed to be less efficient. The possible explanation is that, with the increase of government financial support, hospital directors were encouraged to expand bed size and purchase new equipment regardless of actual need, which may have led to lower utilisation and higher idle cost, and therefore reduced TE. The results showed that sample hospitals with between 228–445 and 446–617 beds, were statistically insignificant regarding TE_CRS_, respectively. However, when hospital beds were over 618, the TE_CRS_ declined. Tan *et al*^56^ explored the optimal scale of secondary hospitals in Beijing, and found that the strict and flexible control standard was 242–353 and 271–571 beds, respectively. Dong *et al*^57^ analysed the optimal scale of county hospitals in Hubei province, and found that the standard was 250–300 beds. Vitaliano^58^ researched the hospitals’ scale economy and indicated that it appears as a shallow U-shaped average cost curve: when hospital beds were beyond an optimum scale, the average cost increased. However, Chinese county hospitals have been experiencing an expansion in bed capacity in recent years, and the average number of beds in sample county hospitals has increased from 373 in 2010 to 462 in 2012. Therefore, directors of hospitals should attach great importance to the expansion of beds and keep hospital beds in an appropriate scale to improve or maintain efficiency. Inefficient hospitals because of large size and those hospitals experiencing DRS should reduce their bed scale.

The ALoS had a negative impact on TE of sample hospitals. The average ALoS was 7.9, which was longer than ALoSs in other countries. For example, in USA, the average ALoS was 5.0.^59^ Therefore, sample hospital managers should adopt necessary measures to reduce ALoS, for instance, implementing clinical pathway management, setting up the monitoring targets, innovating medical technology and spreading the application of ambulatory surgery, etc. The ‘OCCU’ showed a positive impact on TE in this study. In order to improve bed utilisation, the ALoS needs to be shortened to improve bed rotation rate. Moreover, the optimisation of service process is also required. In this study, ‘RONTP’ assumed a positive sign on TE_CRS_. The ‘ROBTN’ also assumed a positive correlation with TE_CRS_, and the average ratio was 2.59. In western countries, it was approximately 0.33.^60^ These gaps between Henan and those abroad may be attributed to the imbalanced allocation of staff and beds, which was mainly caused by the excessive expansion of beds in Henan county hospitals.^55^ In the short term, hospitals may operate with lower human cost (ie, higher RONTP and higher ROBTN) to get higher efficiency, as the Tobit results indicated. However, the imbalanced allocation at the sacrifice of quality may have adverse effects on hospitals in the long run, and has currently deteriorated the relationship between doctors and patients.^60^ Therefore, we propose that sample hospitals should strictly supervise or even reduce hospital beds to coordinate the proportion among physicians, nurses and beds, which will help to guarantee the quality of care and benefit the efficiency eventually.

### Limitations

This study has several limitations. First, the sample county hospitals were selected from Henan province, in central China, while hospitals located in the eastern and western areas were excluded. Second, as with many previous studies, we were temporarily unable to capture the quality of output. In addition, bias adjustments of efficiency and productivity scores were not carried out due to limitation of Coelli's basic DEA approach. In future research, a bootstrap DEA can be employed for more exact results. However, despite its limitations, this study can be considered as a useful preliminary study towards exploring the efficiency, productivity and impact factors in Chinese county hospitals during the period of healthcare reform.

## Conclusions

This study provided an empirical picture of the technical efﬁciency and productivity of county hospitals in healthcare reform. Furthermore, environmental and institutional factors influencing hospital TE were also analysed.

The results indicated a considerable space for technical efﬁciency improvement in Henan county hospitals, since the average TE_CRS_ was, respectively, 0.697, 0.748 and 0.790 for 2010, 2011 and 2012. To operate efficiently, there would be potential for significant input savings in inefficient hospitals. The results of the Malmquist index showed that sample county hospitals experienced a productivity progress of 7.8%, which mainly resulted from the substantial improvement in TC. However, the results implied it might not have created any improvement in hospital governance or management over the period, owing to the deterioration in pure TEC.

The result of Tobit analysis indicated that government subsidy, hospital size with above 618 beds and ALoS were negatively associated with TE; while OCCU, ROBTN and RONTP were significantly positive with TE. So we proposed that county hospitals in Henan should strictly supervise or reduce hospital bed scale; shorten the average length of stay; and coordinate the proportion of physicians, nurses and beds, all of which would benefit the TE.
